# Activated Protein C Resistance Testing: An Update From Australasia/Asia‐Pacific

**DOI:** 10.1111/ijlh.14447

**Published:** 2025-02-19

**Authors:** Emmanuel J. Favaloro, Sandya Arunachalam, Elysse Dean, Mahzuza Salwa, Monica Ahuja, Lynne Connelly, Kent Chapman, Ronny Vong, Leonardo Pasalic

**Affiliations:** ^1^ Department of Haematology, Sydney Centres for Thrombosis and Haemostasis, Institute of Clinical Pathology and Medical Research (ICPMR), NSW Health Pathology Westmead Hospital Westmead New South Wales Australia; ^2^ Faculty of Science and Health Charles Sturt University Wagga Wagga New South Wales Australia; ^3^ School of Medical Sciences, Faculty of Medicine and Health University of Sydney, Westmead Hospital Westmead New South Wales Australia; ^4^ RCPAQAP Haematology St Leonards New South Wales Australia; ^5^ Department of Haematology, NSW Health Pathology St George Hospital Kogarah New South Wales Australia; ^6^ Department of Haematology, NSW Health Pathology Royal North Shore Hospital St Leonards New South Wales Australia; ^7^ Department of Haematology, NSW Health Pathology John Hunter Hospital New Lambton Heights New South Wales Australia; ^8^ Department of Haematology, Institute of Clinical Pathology and Medical Research (ICPMR) NSW Health Pathology, Westmead Hospital Westmead New South Wales Australia; ^9^ Westmead Clinical School University of Sydney, Westmead Hospital Westmead New South Wales Australia

**Keywords:** activated partial thromboplastin time, activated protein C resistance, assay variability, external quality assessment, factor V Leiden, laboratory testing, proficiency testing, Russell viper venom time

## Abstract

**Introduction:**

Activated protein C resistance (APCR) represents a risk factor for thrombosis and is usually due to factor V Leiden (FVL). Clinicians may order either test (i.e., APCR or FVL) to help assess ‘thrombophilia’ in patients who present with thrombosis. APCR testing is usually achieved using clot‐based assays, whereas FVL is assessed by genetic testing. There are advantages and disadvantages to either approach.

**Methods:**

We report updated findings for APCR testing in our geographic region, in part using recent data from the RCPAQAP, an international external quality assessment (EQA) program, with some 50–60 participants for APCR testing over the past decade. Data have been updated to cover the past 13 years (2010–2023 inclusive), with four samples assessed each year, but with a primary focus on new data from 2020 to 2023 inclusive. In addition, data for APCR testing over several years from four large tertiary‐level hospital laboratories have been assessed following a recent change in instrumentation and haemostasis methods.

**Results:**

EQA data continue to show variable performance in both numerical values and their interpretation for APCR testing, with certain methods providing more consistently correct findings than others. In addition, participant interpretation of their own numerical values and transcription errors seem problematic. Finally, the change in recent laboratory testing has also evidenced local improvements.

**Conclusion:**

APCR assays and testing laboratories continue to show variability in performance, with two methods (Pefakit and Staclot) showing the best performance overall. Targeted education may be of benefit, as most of the errors appear to originate from a small proportion of laboratories.

**Abbreviations**
APCR: activated protein C resistance
APTT: activated partial thromboplastin time
DOAC: direct oral anticoagulant
EQA: external quality assessment
FVL: factor V Leiden
RVVT: Russell viper venom time

## 1 Introduction

Activated protein C resistance (APCR) is a well‐known recognised risk factor for thrombosis [1, 2, 3]. APCR can be either congenital or acquired. Most congenital APCR is due to factor V Leiden (FVL), caused by a mutation in the *FV* gene. Activated protein C (APC) normally acts to cleave activated FV (and also FVIII) (i.e., FVa, FVIIIa); however, the mutation causing FVL is resistant to cleavage by APC, and hence leads to APCR.

APCR testing is generally achieved using clot‐based assays, whereas FVL assessment requires genetic evaluation of the *FV* gene [1, 2, 3, 4, 5]. Clinicians may order either APCR or FVL testing in patients being investigated for a recent thrombosis (i.e., perceived ‘thrombophilia’). There are advantages and disadvantages to either approach. APCR testing has the advantage of being able to be performed on most automated haemostasis analysers, with most major haemostasis suppliers also providing APCR reagents. In addition, APCR testing can identify both congenital APCR (e.g., due to FVL or other *FV* variants), and acquired APCR, either of which may yield a procoagulant phenotype. On the other hand, APCR assays have variable sensitivity to FVL, to the different causes of acquired APCR (i.e., elevated FVIII will not affect non‐APTT assays, pre‐dilution assays tend to compensate for most causes of acquired APCR) [5], and also to various clinically used anticoagulants, which may cause problematic interference in APCR testing that may lead to either false positive or false negative APCR findings [6, 7, 8, 9, 10]. FVL testing has the advantage of being unaffected by clinical anticoagulants, and of providing a discrete outcome (i.e., wild type, or heterozygous or homozygous for FVL), with false positives and negatives being rare [11]. Genetic testing can also identify pseudo‐homozygosity for FVL [12, 13]. The disadvantages of FVL testing include limited availability, usually higher cost than APCR, and potential insurance implications of having identified a confirmed genetic ‘mutation’ in any individual [14]. In addition, FVL testing cannot inform on acquired APCR and depending on methodology may not detect other *FV* variants which cause APCR (e.g., Factor V Hong Kong or Factor V Cambridge) [2].

In the current report, we assess the utility of APCR testing via two approaches. First, we provide an update of APCR methods and test results from a large external quality assessment (EQA) program, with data now available for the period 2010–2023 inclusive. In a separate analysis, we assess how a change in test methodology has improved the ability of laboratory APCR testing to identify FVL in the age of direct oral anticoagulants (DOACs).

## 2 Materials and Methods

### 2.1 EQA Setting

This study in part assesses data submitted by participants of the Royal College of Pathologists of Australasia Quality Assurance Program (RCPAQAP) in Haematology (https://rcpaqap.com.au/). These data therefore represent ‘real world’ assessments of laboratories accredited to perform APCR testing, although a variety of methods are in use, and these have potentially variable utility. The RCPAQAP despatches 4 samples per year to participants, with these being either normal (or negative for APCR) or else representing APCR positive samples. Data from 2010 to 2023 inclusive, representing the last 13 years of complete test data have been presented for trends, with this in part representing an update from a recent past report [15]. However, the majority of the report presents new data from 2020 to 2023, representing the last 4 years of complete data. Current participants for the APCR module mostly derive from Australia (*n* = 24), with additional representation from New Zealand (*n* = 3), Hong Kong (*n* = 3), Malaysia (*n* = 3), India (*n* = 3), South Africa (*n* = 2), Columbia (*n* = 2), France (*n* = 1) and Austria (*n* = 1).

### 2.2 EQA Samples

APCR samples are generated in‐house from bags of fresh frozen plasma (FFP), derived from the Australian Red Cross Blood Bank, with this material providing sufficient plasma to create several duplicate samples to be sent in different years. The FFP samples represent single human plasma donations and were screened for the presence of APCR using the Siemens ProC Ac R assay (see Table S1). FFP samples identified to have a low APCR ratio consistent with APCR were used as APCR ‘positive’ samples. FFP samples identified to have a normal APCR ratio inconsistent with APCR were used as APCR ‘negative’ samples or in another EQA module. All APCR module samples (i.e., either APCR ‘positive’ or APCR ‘negative’) are delivered to participants as lyophilised samples as 1.0 mL/vial. For the APCR module, four samples are sent per year as paired samples for two separate proficiency challenges, spaced out over a 12‐month period. The samples are sent in a semi‐random fashion, and duplicates are sent over different surveys to help assess comparability or reproducibility between surveys.

### 2.3 EQA Program Aims and Strategy of Sample Dispatches

For APCR testing, the major aim of the RCPAQAP is to provide laboratories with material that enables the RCPAQAP to assess and hopefully ensure testing precision and accuracy for APCR. This process therefore provides independent external evaluation of a laboratory's performance. The dispatch plan incorporates APCR positive (i.e., as identified using the Siemens ProC Ac R assay) and normal (i.e., APCR negative samples again using the Siemens ProC Ac R assay) to identify potential risks of reporting incorrect results and minimising potential harm to patients and reputational damage to laboratories.

### 2.4 EQA Data Analysis

Numerical values for APCR testing are submitted electronically by all participants for the tests they perform. This data capture therefore attempts to identify the normal test practice for each participant. Participants are also required to interpret their own test findings in terms of APCR being present or not. Specifically, participants may report ‘normal’ or ‘negative [for APCR]’, or ‘reduced’ or ‘positive [for APCR]’, depending on the sample, participant preference and method in use (including product information guidance). Participants can supplement their reported findings with comments to explain issues with testing or further action to be taken.

Due to variable reporting among participants, limited test numbers for individual methods, and sometimes high numerical variability, participant‐reported numerical values for APCR testing are not assessed by the RCPAQAP for individual participant reports, as they would require very wide allowable limits of performance (ALP); nevertheless, numerical values are reported to participants and plotted in RCPAQAP generated participant reports against peer group values for participants to self‐assess their numerical results. Instead, for this EQA module, the RCPAQAP assesses participant interpretation of their own numerical values, as this is the more clinically relevant parameter (i.e., clinicians want to know if their patient has APCR or not). The RCPAQAP assesses participant interpretations as being either concordant or not concordant to the ‘target’ interpretation. The RCPAQAP requires participant responses to achieve ≥ 80% consensus to enable the characterisation of a valid target interpretation and the subsequent assessment of participant responses as concordant or not concordant. If < 80% consensus is achieved, this is designated as not assessed. This rarely occurs in practice, since ≥ 80% consensus is usually achieved for APCR testing in this module, and indeed was achieved for all samples assessed between 2020 and 2023 inclusive.

### 2.5 Laboratory Audit

In addition to the EQA evaluation, we show recent data for a laboratory audit of APCR testing. Four large hospital tertiary level units from NSW Health Pathology (NSWHP) contributed data to this audit, comprising data available to them for combined ACPR and FVL testing over the past 2–4 years.

### 2.6 Ethics

Samples utilised by the RCPAQAP for the APCR module represent normal human donor FFP, for which donors agree can be utilised in research or quality control. As this report otherwise represents a review of EQA practice and of laboratory test practice (or essentially a quality audit), not otherwise identifying any patients or patient‐centric information, specific Ethics approval for the study was not deemed to be required.

## 3 Results

### 3.1 Evolution in Test Methodologies and EQA Participant Numbers

**FIGURE 1:**
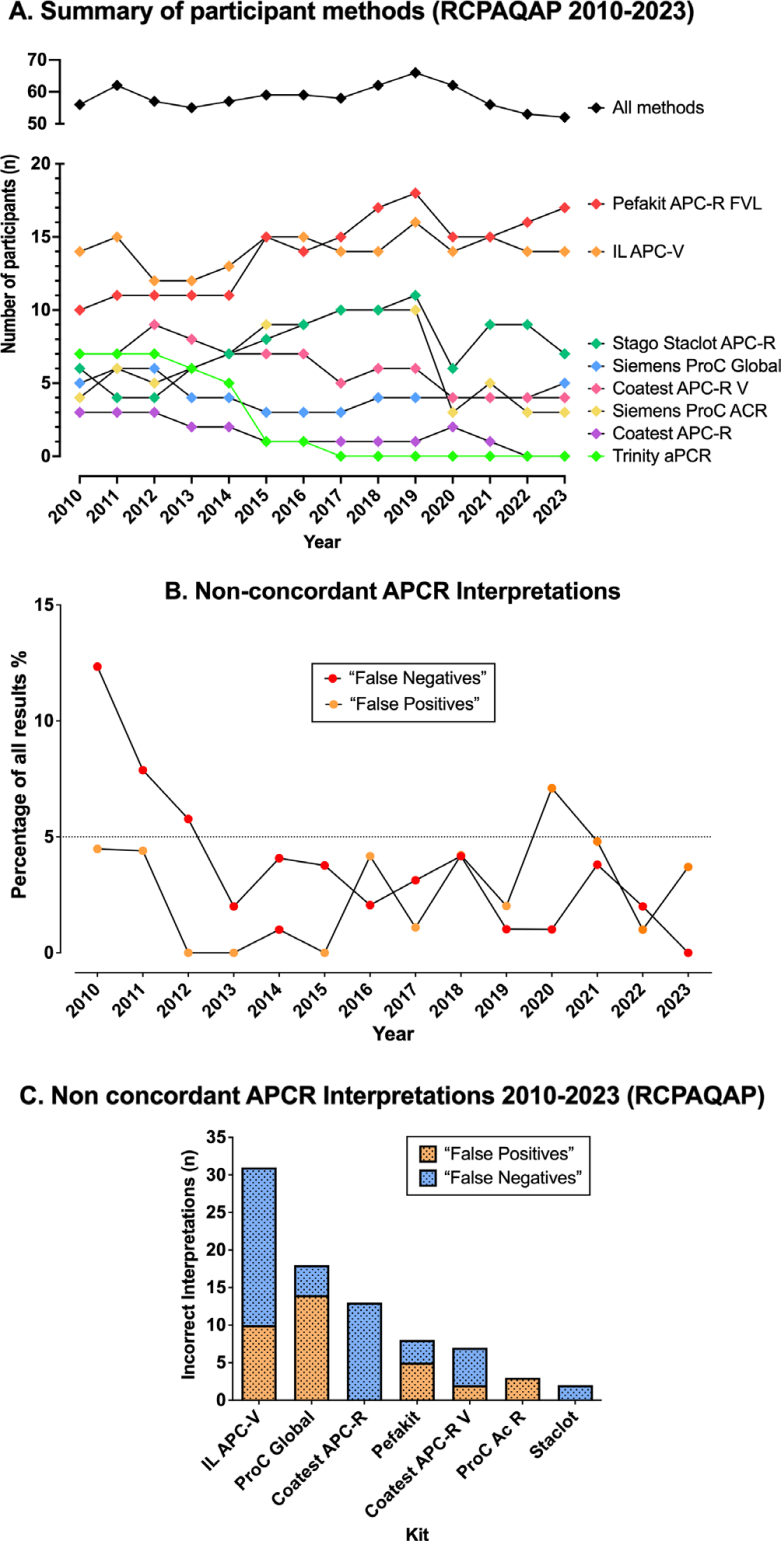
Changes in participant numbers and APCR methods used by RCPAQAP participants (2010–2023 inclusive), and summary of APCR interpretation errors. (A) Participant submitted results for APCR testing by year (2010–2023 inclusive). Several participants submit results for more than one instrument; hence these numbers are higher than total participant numbers. (B) Non‐concordant APCR interpretations by participants by year (2010–2023 inclusive) and representing false positives (laboratory indicated ‘reduced’ or ‘positive’ for normal samples) or false negatives (laboratory indicated ‘normal’ or ‘negative’ for an APCR positive sample). (C) Non‐concordant APCR interpretations by participants by year (2010–2023 inclusive) by method. Full method names/manufacturers are provided in Table S1. This data extends those of a recent publication [15].

Full test names and manufacturers for the APCR assays utilised by participants are given in Table S1. Participation numbers for EQA testing have decreased slightly over recent years for APCR testing (Figure 1A). It is unclear why this is so, but may reflect an increasing adoption of, or preference for, direct genetic testing for FVL. Testing using some methods has reduced (e.g., Chromogenix Coatest), whereas others have remained steady (e.g., Werfen IL APC‐V) and others have increased (e.g., Pefakit APC‐R FVL, Stago StaClot APC‐R) (Figure 1A).

### 3.2 Participant Interpretations

In addition to providing numerical test data, RCPAQAP participants are required to provide an interpretation of whether their own test results are consistent with the presence of APCR or not. Depending on the methodology used, and any associated product information guidance, participants may report either ‘normal’ or ‘negative [for APCR]’ for normal samples or they may report ‘reduced’ or ‘positive [for APCR]’ for APCR samples. Summary data for errors in interpretation (when a laboratory indicates ‘normal or negative’ for an APCR sample or ‘reduced or positive’ for a normal sample) and representing the last 13 years of data, thereby extending data from a prior report [15], are shown in Figure 1B, with additional new information regarding interpretation errors for the period 2020–2023 detailed in Table 1. Most participants (typically > 95%) correctly identified most samples (Figure 1B) as being ‘normal or negative’ (for a normal sample) or ‘reduced or positive’ (for an APCR sample); of interest, false negative error rates have fallen over time, whereas false positive error rates seem to have in general not improved over time, although both types of errors currently remain < 5% overall.

**TABLE 1:** Summary of interpretation errors for samples despatched in 2020–2023 inclusive.

Sample ID	Lab no	False pos using	Ratio	Comment
20‐02	1	IL APC‐V	2.47	Normal ratio; probable participant transcription error; also reported reduced for sample 20‐01
	1	IL APC‐V	2.54	Normal ratio; probable participant transcription error; also reported reduced for sample 20‐01
	8	Pefakit	4.55	Normal ratio; probable participant transcription error; also reported reduced for sample 20‐01
	9	Pefakit	5.7	Normal ratio; probable participant transcription error; also reported reduced for sample 20‐01
	2	SM Pro C Global	0.62	Low relative ratio reported; also reported reduced for sample 20‐01
	10	SM Pro C Global	2.1	Ratio near cut‐off; also possible participant interpretation or transcription error; also reported reduced for sample 20‐01
20‐04	2	SM Pro C Global	0.62	Low relative ratio reported; also reported reduced for sample 20‐03
21‐01	3	SM Pro C Global	1.54	Low ratio; probable transcription error; also incorrectly reported normal for sample 20‐02, and appears results reversed
	4	IL APC‐V	1.68	Low ratio; probable transcription error; also incorrectly reported normal for sample 20‐02, and appears results reversed
	2	SM Pro C Global	0.62	Low relative ratio reported; also reported reduced for sample 20‐01
	11	IL APC‐V	2.49	Low relative ratio reported; also reported reduced for sample 20‐01
21‐03	2	SM Pro C Global	1.89	Low ratio; also reported reduced for sample 20‐04
22‐02	5	IL APC‐V	2.4	The reported result was a normal ratio, but the participant reported the interpretation as reduced; possible transcription error for interpretation since participant comments also indicated the result was within the normal range, and also reported a reduced ratio for sample 22‐01 (for the ratio of 1.71)
22‐04	5	SM Pro C Global	1.97	False low ratio
23‐02	6	IL APC‐V	1.99	False low ratio; 2.2 noted as cut‐off; same lab as below
	6	IL APC‐V	2.01	False low ratio; 2.2 noted as cut‐off; same lab as above
23‐04	12	Pefakit	0.16	False very low ratio; transcription or calculation error?

Sample ID	Lab no	False neg using	Ratio	Comment
20‐01	3	SM Pro C Global	1.21	Low ratio; probable participant interpretation or transcription error; also reported normal for sample 20‐01
21‐02	3	SM Pro C Global	2.28	Normal ratio; probable transcription error; also incorrectly reported reduced for sample 20‐01, and appears results reversed
	4	IL APC‐V	2.46	Normal ratio; probable transcription error; also incorrectly reported reduced for sample 20‐01, and appears results reversed
21‐04	4	IL APC‐V	1.83	Low ratio with added FV def pl., but reported normal ratio without added FV def pl. (2.39)
	7	Coatest	2.53	False normal ratio
22‐03	7	IL APC‐V	2.7	False normal ratio; cut off noted as 2.5

Of additional interest, certain methods appear to be associated with greater numbers of false positive or false negative interpretations for APCR than other methods (Figure 1C). Considering participant numbers using particular methodologies (Figure 1A), the Werfen IL APC‐V and Chromogenix Coatest methods, both APTT‐based methods, were associated with the greatest number of errors, and the Pefakit APC‐R FVL and Stago StaClot APC‐R with the least number of errors. The reduction in false negative rates (Figure 1B) over time may be in part due to method changes (i.e., reduced use of Coatest APC‐R assay, previously responsible for many false negative events [15] (Figure 1A)).

However, we could also identify problems with particular laboratories, such that a few laboratories seemed to be associated with most of the errors. These data are summarised in Table 1. A total of 23 errors (17 false positive APCR, 6 false negative APCR) in interpretation were reported by 12 laboratories over the period 2020–2023 inclusive. Seven laboratories were responsible for 18 errors. Several errors appeared to be transcription‐related, rather than method‐related, with reversed results of paired samples sent in particular surveys.

### 3.3 Numerical Reported Values

**FIGURE 2:**
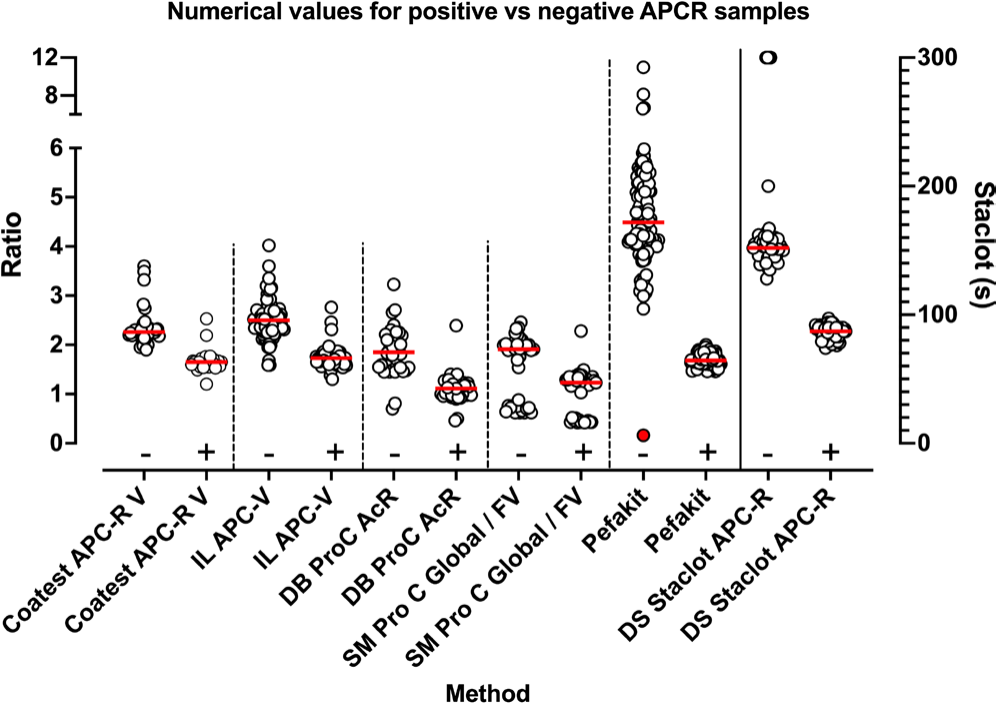
Numerical values for positive versus negative APCR samples (2020–2023 inclusive). APCR ratio shown on the left y‐axis for all methods except Staclot, for which results are shown on the right axis in seconds. The x‐axis indicates the sample status as APCR negative (−) or positive (+). Most methods show an overlap in APCR ratios between APCR – and + samples, and some participants reported lower ratios than those of most peer group for Siemens methods. In contrast, clear separation of APCR negative (−) or positive (+) samples was only seen for the Stago Staclot method, and also for the Pefakit method (excluding a single reported result of 0.16 [red symbol], and reflecting an obvious outlier and presumed reporting error). Red horizontal bars indicate method median values.

Although not formally assessed by the RCPAQAP in participant reports, numerical values reported by participants for similar methodologies were, in general, similar (Figure 2; only the most recent years 2020–2023 are shown). However, most methods appeared to yield an overlap of numerical data between APCR negative vs. positive samples. Indeed, complete separation of this dichotomous data was only seen for two methods, Pefakit (excluding one gross outlier value, Figure 2 red symbol) and Stago APC‐R. In addition, some participants reported much lower APCR ratios than their peer group values for Siemens methods (Figure 2). These outlier points were derived from specific participants and we could not identify the reasons for these outliers; several of these participants were also responsible for several errors listed in Table 1.

### 3.4 Reproducibility of Test Results Using Repeat Test Samples

**FIGURE 3:**
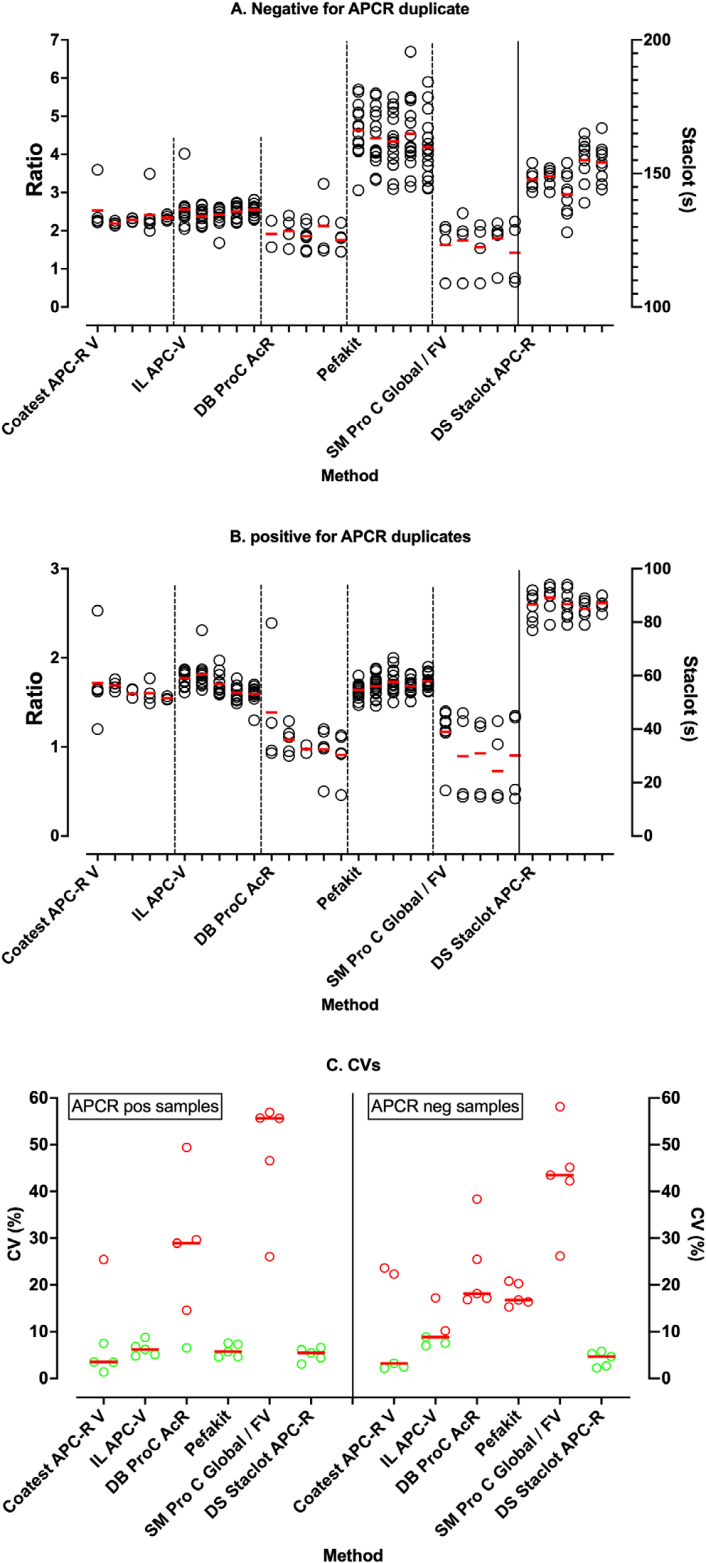
Results of testing of duplicate samples for APCR in different survey despatches for different methods. APCR ratios shown on the left y‐axis for all methods except Staclot, for which results are shown on the right axis in seconds. Red horizontal bars indicate method median values. (A) One sample negative for APCR tested on multiple surveys (20‐02, 20‐04, 21‐01, 21‐03, 22‐02). (B) One sample positive for APCR tested on multiple surveys (21‐04, 22‐01, 22‐03, 23‐01, 23‐03). (C) Coefficient of variation (CV) data for data shown in A and B.

As noted, repeat identical samples are sent to participants in different despatches. In general, numerical data returned by participants showed similar means and ranges for repeat sample testing, with examples shown in Figure 3, as separated into repeat testing for one normal (APCR negative) sample (Figure 3A) and one APCR positive sample (Figure 3B). This essentially indicates generally good within the laboratory or within method reproducibility or consistency and also acts as a surrogate marker of sample stability. Nevertheless, we also note some variation in absolute test values; on occasion, these appear as outlier points (e.g., Coatest data, Siemen's methods), and on occasion generally wide value ranges (e.g., Pefakit for normal sample). This variation is also presented in Figure 3C, which shows the calculated coefficient of variation (CV; %) values corresponding to the data shown in Figure 3A,B. CVs were in general higher for the normal sample, and generally under 10% for the APCR positive sample for all methods except the Siemen's assays, which is likely due to the outlier points.

### 3.5 Dabigatran Interference in APCR Testing and Use of DOAC Stop

During the assessment period, the RCPAQAP despatched a normal sample containing dabigatran (sample 24‐03). Participants were not informed that the sample contained dabigatran, which was assessed as being 228 ng/mL. Exactly 17 out of 43 enrolled participants correctly identified and reported DOAC interference in this sample. Six (of 17) participants were using the Pefakit and treated the sample with either DOAC Stop (*n* = 5) or another source of activated charcoal (*n* = 1) and all six were subsequently able to report a normal ratio (range 5.1–8.1) and a normal interpretation. The remaining 11 participants did not treat the sample with any DOAC neutralizer; only two of these were able to provide a (normal) numerical value and a normal interpretation (the remaining 9 participants [8/9 using Pefakit] were unable to provide a valid interpretation for the sample because of the DOAC interference). This indicates that the Pefakit may not provide reliable APCR data in the presence of dabigatran interference, but that treatment with a DOAC neutralizer can remove this interference and provide reliable results/interpretation.

### 3.6 Result of Laboratory Audits: APCR vs. FVL

**FIGURE 4:**
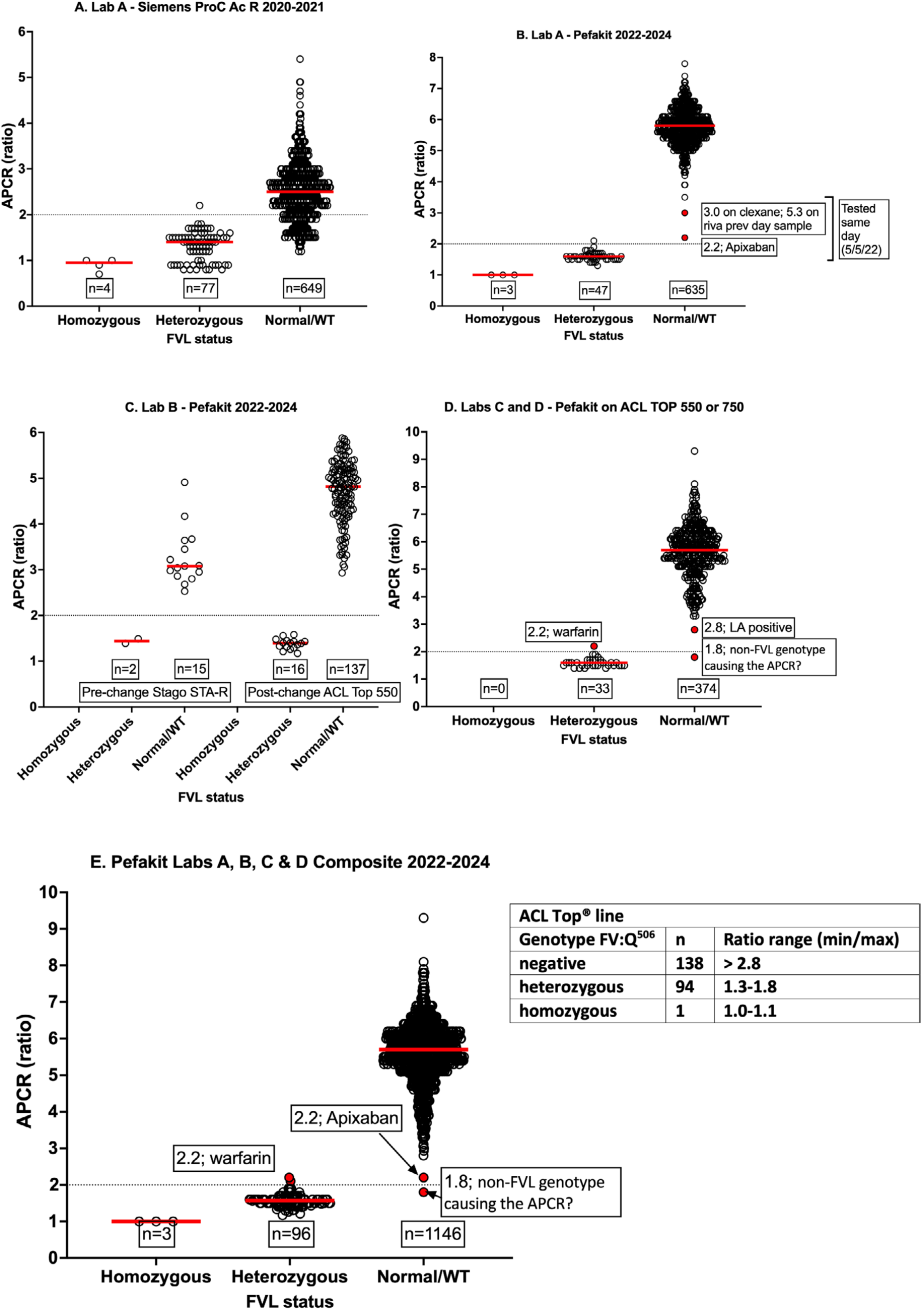
An audit of APCR versus FVL testing in four laboratories. APCR ratio was shown on the y‐axis and FVL status on the x‐axis. (A) Lab A, when using the Siemens ProC Ac R method on Stago STAR analyser (2020–2021 inclusive data). (B) Lab A, when using the Pefakit method on ACL Top 750 analyser (2022–2024 to date). Two samples for patients on clexane (switched from rivaroaxaban) and apixaban yielded unexpectedly low ratios for normal/wild‐type *FV* on a single day of testing. (C) Lab B, when using the Pefakit method on Stago STAR analyser or ACL Top 750 analyser (2022–2024 [to date]). (D) Labs C and D, when using the Pefakit method on an ACL Top 550 or 750 analyser. (E) Composite of data from Labs A, B, C and D for Pefakit (2022–2024 [to date]). The manufacturer‐recommended ranges are shown in the side Table.

Four laboratories audited their patient tested and reported data for APCR and FVL as available to them. Figure 4 shows data for patients where both APCR and FVL testing were performed. In Lab A, a prior methodology was based on the Siemens ProC Ac R method (Figure 4A) assessed on a CS5100 instrument (Siemens, USA). Although normal/wild type (WT) *FV* samples in general yielded higher APCR ratios than FVL positive cases, there was an overlap with FVL heterozygous samples, so no clear discrimination was possible. In Lab A, after a change of instrumentation (to ACL TOP 750; Werfen, USA) and methodology (to Pefakit), there was much clearer separation of APCR groups, with overlap of only two samples, potentially due to anticoagulant interference (Figure 4B). In Lab B, there was no change in method, but only a change in the instrument (from STAR Evolution [Stago, France] to ACL TOP 550 [Werfen, USA]). In both situations (i.e., pre‐ and post‐instrument change), the Pefakit data show a clear separation of APCR ratios between wild‐type and FVL heterozygous patients (Figure 4C). Data for Labs C and D is only available for post‐instrument change to ACL Top 550 or ACL Top 750 (Figure 4D), with general discrimination between normal/wild type and FVL heterozygous patients, except for a few overlapping data points. Figure 4E shows the composite of data for the Pefakit method and FVL status from all laboratories, alongside the manufacturer‐recommended ranges. Using a dataset exceeding 1200 samples, the manufacture ranges can be considered validated.

## 4 Discussion

This contemporary assessment of APCR testing practice from participants of the RCPAQAP provides a contemporary evaluation of current APCR testing in ‘real world’ laboratories. In part, the data reflect an update of another report from the RCPAQAP in 2020 in regard to test method trends (Figure 1) [15]. The summarised data for the last 13 years have now been assessed for a large number of samples (*n* = 52), which have been cross‐laboratory assessed in up to 60 instruments, depending on data year (Figure 1A), and in total representing over 2500 participant responses. The remaining data is new and reflect RCPAQAP findings from 2020 to 2023 (Figures 2 and 3, Table 1), representing the most recent complete year data sources, as well as a multi‐centre audit of APCR test practice (Figure 4) after a changeover in methodology to Pefakit in 2020 [16].

There are several elements of our findings that are worth discussing. First, although overall participant numbers have only reduced slightly over the past 14 years, the landscape has certainly shifted in terms of methods in use, with the disappearance of two methods (Trinity aPCR and Coatest APC‐R), and the current predominance of two methods (Pefakit and IL APC‐V) (Figure 1A). In line with this trend, the number of false negatives reported by participants has decreased (Figure 1B), most likely due to the reduction in Coatest APC‐R usage, given this method generated many false negative interpretations in the past [15] (Figure 1C). The residual error rate is now consistently below 5% (Figure 1B), meaning that the vast majority of participants (i.e., > 95%) can be identified as providing satisfactory performance for APCR testing. Of relevance, many of the residual interpretation errors are not due to methodology per se, but instead to misinterpretation by some laboratories (Table 1), indicating that further education is key. Indeed, several interpretation errors related to Pefakit and StaClot methods (Figure 1C) were likely transcription errors (Table 1 and reference [15]), rather than method failures. The reason for method failures for other methods appears to be the inability to clearly discriminate between APCR positive and APCR negative cases, with considerable overlap of numerical data. Thus, clear separation of data points is only seen with Pefakit and StaClot methods (Figures 2 and 4), with all other methods showing substantive overlaps in data points (Figure 2), indicating poor discrimination or specificity of these assays for APCR.

Overall within method variation (CVs) was also a concern (Figure 3C), and differed according to method and whether samples were APCR positive or negative. This helps validate our decision to not assess these values as part of the ongoing participant assessment process, since it would require the establishment of varied, and likely very wide, limits of acceptability, according to method and sample type. The lowest overall CVs were for the StaClot method, which were < 10% for both APCR negative and positive samples. This may be because the method is instrument specific for the Stago range of analysers. Instead, the Pefakit is not restricted to a single type or range of analysers and can be performed on any modern haemostasis analyser, thus potentially explain the higher CVs for the normal samples.

Regarding local APCR practice in the NSWHP group, data (Figure 4) have clearly validated the use of the Pefakit on the network's current range of ACL TOP (Werfen) analysers, and indeed also validated our decision to not accept the APCR solution offered by Werfen (IL APC‐V) [16]. Despite the growth of DOAC use in Australia [17], and the risk of false positive and negative thrombophilia events that may arise [10], the Pefakit appears to be mostly unaffected, except for dabigatran, which is now not utilised so heavily in Australia [17].

### 4.1 Literature Review and Comparison With Other EQA and Published Findings

As part of this evaluation, we also performed various PubMed searches, with one using the search string ([activated protein C resistance OR APCR]) AND ([external quality assessment] OR [external quality assurance] OR [proficiency testing]) (performed on 20 November, 2024). This search only returned 8 citations, which were then screened for relevance. Some citations were unrelated to APCR testing. From those potentially relevant (i.e., related to EQA practice for APCR), Hollestelle et al. performed a recent systematic review and meta‐analysis of within‐subject and between‐subject biological variation data of coagulation and fibrinolytic measurands including APCR [18]. They reported overall low within‐subject and between‐subject biological variation data for APCR but did not provide information on reagents used for these studies. Indeed, such data are likely attributable to certain methods, and perhaps not generalisable to all methods, nor directly comparable to data in our report. Lindahl et al. reported on dabigatran interference in two APCR assays, namely the Coatest and Pefakit assays, showing concentration‐dependent prolongation of APCR ratios for both assays [19]. Several articles were published in the years 2005–2007, including from us, and noting past EQA test performance data for APCR [20, 21, 22, 23, 24]. For RCPAQAP data, we reported approximately equal numbers of laboratories performing APTT‐based or RVVT‐based APCR assays, but that RVVT‐based assays were usually better, with fewer error rates [20, 21, 24]. Even then, we noted that the APTT method group yielded higher false‐negative and/or false‐positive findings (in ~5% occasions), which is similar to the data presented in the current report. Also, at that time, most laboratories using APTT‐based APCR assays were using predilution in FV‐deficient plasma, whereas most laboratories using RVVT‐based APCR assays were not. Thus, it appears that the current article reflects the latest update on APCR test performance EQA data available in the literature. Notably, the years 2005–2007 were prior the release of the DOACs, which now cause unwanted interference in all clot‐based assays, including APCR [6, 7, 8, 9, 10, 25].

In the Westmead laboratory, we historically utilised the Siemens ProC Ac R assay, which we found to provide excellent discrimination of FVL status in the years before DOACs [26], and this test was therefore used to screen for APCR in FFP samples used for the RCPAQAP APCR module. In an article published in 2020, we reported on a study using rivaroxaban to investigate interference in APCR assays, and the ability of DOAC Stop to remove the associated interference [8]. Best performance was achieved using the Pefakit, Coatest APC‐R V and Stago APCR assays. However, the Pefakit can be affected by dabigatran, which we also noted in a recent evaluation aiming to decide which APCR assay to adopt in our laboratory network [16]. We again show in the current report that dabigatran can cause interference in this assay in the current data using sample 24‐04, and that DOAC neutralisation with agents such as DOAC Stop can eliminate this interference. Others have also noted dabigatran interference in this assay [27] and this interference is also noted in the product information [28]. Our Laboratory Network eventually agreed to employ the Pefakit assay, as this assay appeared insensitive to both rivaroxaban and apixaban [16], with these DOACs representing the most often used oral anticoagulants in Australia [17]. Indeed, dabigatran use in Australia is now very low [17]. The manufacturer notes the interference of both dabigatran and argatroban in the assay since the assay uses a prothrombin activator [28]. Also, the Pefakit uses a factor V activator, not a factor X activator, thereby making the assay fairly insensitive to interference from apixaban and rivaroxaban. Finally, in the current study, the manufacturer's recommended ranges could be adopted for use (Figure 4E). We only identified a few outlier data points (Figure 4E), including two patients on anticoagulant therapy (warfarin, apixaban), although these should not cause interference in the assay. It is likely that such samples represent complex and rare samples, and it may be safe to reflex to genetic testing for FVL in any patient where APCR results may be unreliable for any given method. For example, a FV mutation other than FVL could feasibly have been responsible for the APCR ratio of 1.8 in the FVL negative group.

### 4.2 Study Limitations

There are various study limitations that we should highlight. First, samples were prepared in‐house for this EQA module, as derived from normal human donor FFP, and as assessed for APCR by screening using a single APCR method (Siemens ProC Ac R assay). No genetic testing for FVL status is possible on FFP, and so we do not know the cause of the APCR in the FFP samples, and so we cannot assert that these samples actually reflect FVL status. Although FFP represents human plasma, FFP samples may not accurately reflect identically with native patient plasma being assessed for APCR. This may also lead to some possibility of ‘non‐commutability’ of the sample material used [29]. The RCPAQAP, like other EQA providers, cannot source adequate volumes of patient plasma from patients with APCR required for EQA use (e.g., for 60 instruments, with 1 mL despatches, as duplicate samples, with homogeneity and stability testing, would require > 150 mL plasma, or a > 300 mL blood collection). This would need to be expanded × 2 for 1 year of EQA for each sample. There are also Ethical issues related to the use of patient samples for EQA, where alternate samples may suffice. Ethical issues include: (i) lack of direct relationship between EQA providers and potential patient donors, and thus potential access to private personal health information; (ii) non‐equal relationships of caregivers and patients should clinicians request donations from patients on behalf of the EQA and (iii) requirement for appropriate staff and facilities to enable large blood collections in an ethically responsible manner.

In addition, the limited number of participants performing APCR assays with a particular method means that study numbers are inherently limited for each method.

Thus, the evaluation of findings from EQA needs to be accordingly moderated. For example, we cannot fully identify that certain assays appear to provide more accurate APCR findings than other assays, but we can identify that the Pefakit and Stago assays were consistently the best performers in this EQA. This is despite using a different assay (ProC Ac R) to screen samples for APCR ahead of despatch. In addition, most laboratories (> 95%) correctly identified the presence or absence of APCR in most samples, and most of the errors appeared to derive from a small group of laboratories (Table 1).

Of additional relevance, we could also show that the Pefakit used in a real‐world setting, including patient samples derived from patients on anticoagulants, still provides for good discrimination of FVL status (Figure 4). Finally, since EQA data are collated as reported by RCPAQAP participants, and sometimes errors are noted to occur (Table 1), the reported variation in test values reflected by EQA CVs is likely to be higher than those reflective of assay variation per se.

## 5 Conclusion

To our knowledge, we report the most comprehensive and contemporary evaluation of EQA findings related to APCR testing. Although we recognize limitations in the use of EQA to inform APCR testing, data generally identifies continued ongoing good performance with certain methods. These findings are encouraging and should provide some reassurance to laboratories undertaking these tests. We also suggest that laboratories continuing to use some less well‐performing tests reconsider if they remain relevant in the age of DOAC interference.

## Author Contributions

All authors contributed to various elements of the study execution, study design, or data analysis. In particular, E.J.F., S.A., E.D. and M.S. are responsible for the RCPAQAP elements of the study, and E.J.F., M.A., L.C., K.C., R.V. and L.P. are responsible for the APCR audit data (Figure 4). E.J.F. wrote the original draft of the manuscript, which was then revised according to input from all other contributors; All authors approved the manuscript for submission and publication.

## Conflicts of Interest

The authors declare no conflicts of interest.

## Supporting information


Table S1.

## Data Availability

The data that support the findings of this study are available from the corresponding author upon reasonable request.
