# Supplementary material for: Activated Protein C Resistance Testing: An Update From Australasia/Asia‐Pacific
Source: Int J Lab Hematol. 2025 Feb 19;47(4):720–9. doi: 10.1111/ijlh.14447 (PMC12239697; doi:10.1111/ijlh.14447)
Supplement: Supplementary file 1 — Table S1. [file IJLH-47-720-s001.docx]

**Supplementary Table 1.** Summary of APCR assays used by RCPAQAP participants.*

| **Assay** | **Manufacturer** |
| --- | --- |
| COATEST™ APC™ Resistance V | Chromogenix (Werfen, Instrumentation Laboratory Company, Bedford, MA, USA) |
| Pefakit® - APC-R Factor V Leiden | Pentapharm, Parsippany, NJ, USA |
| STA-Staclot APC-R | Diagnostica Stago S.A.S. Asnières sur Seine Cedex France |
| Factor V Leiden (APC™ Resistance V) | Werfen, Instrumentation Laboratory Company, Bedford, MA, USA |
| Protein C [ProC®] Global Assay | Siemens Healthineers,  Siemens Healthcare GmbH, Erlangen, Germany |
| ProC Ac R | Siemens Healthineers,  Siemens Healthcare GmbH, Erlangen, Germany |

APCR, activated Protein C resistance; RCPAQAP, Royal College of Pathologists of Australasia Quality Assurance Program.

All manufacturers have various international addresses; addresses given represent international headquarters.
